# Construction of Novel Methylation-Driven Gene Model and Investigation of PARVB Function in Glioblastoma

**DOI:** 10.3389/fonc.2021.705547

**Published:** 2021-09-10

**Authors:** Wanli Yu, Pengfei Wu, Fang Wang, Li Miao, Bo Han, Zhiqun Jiang

**Affiliations:** ^1^Department of Neurosurgery, Gaoxin Hospital of the First Affiliated Hospital of Nanchang University, Nanchang, China; ^2^Department of Neurosurgery, The First Affiliated Hospital of Nanchang University, Nanchang, China; ^3^Department of Neurosurgery, The First Affiliated Hospital of University of Science and Technology of China (USTC), Division of Life Sciences and Medicine, University of Science and Technology of China (USTC), Hefei, China; ^4^Anhui Key Laboratory of Brain Function and Diseases, Hefei, China; ^5^Department of Neurosurgery, The Second Affiliated Hospital of Harbin Medical University, Harbin, China; ^6^Central Laboratory, Gaoxin Hospital of the First Affiliated Hospital of Nanchang University, Nanchang, China; ^7^Department of Neurosurgery, Beijing Tiantan Hospital, Capital Medical University, Beijing, China

**Keywords:** glioblastoma, methylation-driven genes, biomarker, prognostic indicators, PARVB, EMT

## Abstract

**Background:**

Glioblastoma multiforme (GBM) is characterized by widespread genetic and transcriptional heterogeneity. Aberrant DNA methylation plays a vital role in GBM progression by regulating gene expression. However, little is known about the role of methylation and its association with prognosis in GBM. Our aim was to explore DNA methylation-driven genes (DMDGs) and provide evidence for survival prediction and individualized treatment of GBM patients.

**Methods:**

Use of the MethylMix R package identified DMDGs in GBM. The prognostic signature of DMDGs based on the risk score was constructed by multivariate Cox regression analysis. Receiver operating characteristics (ROC) curve and C-index were applied to assess the predictive performance of the DMDG prognostic signature. The predictive ability of the multigene signature model was validated in TCGA and CGGA cohorts. Finally, the role of DMDG β-Parvin (PARVB) was explored *in vitro*.

**Results:**

The prognostic signature of DMDGs was constructed based on six genes (*MDK*, *NMNAT3*, *PDPN*, *PARVB*, *SERPINB1*, and *UPP1*). The low-risk cohort had significantly better survival than the high-risk cohort (p < 0.001). The area under the curve of the ROC of the six-gene signature was 0.832, 0.927, and 0.980 within 1, 2, and 3 years, respectively. The C-index of 0.704 indicated superior specificity and sensitivity. The six-gene model has been demonstrated to be an independent prognostic factor for GBM. In addition, joint survival analysis indicated that the *MDK*, *NMNAT3*, *PARVB*, *SERPINB1*, and *UPP1* genes were significantly associated with prognosis and therapeutic targets for GBM. Importantly, our DMDG prognostic model was more suitable and accurate for low-grade gliomas. Finally, we verified that PARVB induced epithelial-mesenchymal transition partially through the JAK2/STAT3 pathway, which in turn promoted GBM cell proliferation, migration, and invasion.

**Conclusion:**

This study demonstrated the potential value of the prognostic signature of DMDGs and provided important bioinformatic and potential therapeutic target data to facilitate individualized treatment for GBM, and to elucidate the specific mechanism by which PARVB promotes GBM progression.

## Introduction

Glioblastoma multiforme (GBM) is the most frequent primary brain tumor in adults. Despite aggressive treatment regimens, GBM patients generally have a poor prognosis of less than 14 months (1). Rapid development in bioinformatics has greatly contributed to explorations of the molecular characteristics of cancer. As well, specific GBM molecular markers have been discovered, providing new insights into the progression mechanism, diagnosis, and treatment of GBM (2). Among the many molecular markers, isocitrate dehydrogenase (IDH)1/2 mutation and O^6^-methylguanine DNA methyltransferase (MGMT) methylation are the best-known. Compared with GBM patients without IDH mutations, GBM patients with IDH mutations have longer survival times (3, 4). GBM patients with MGMT methylation are more sensitive to temozolomide (TMZ) (5, 6). Therefore, it is clinically important to identify effective and promising biomarkers for the prognosis of GBM.

DNA methylation modification is an important part of epigenetics, which contributes to the transcriptional regulation of genes and maintenance of genomic stability (7). DNA methylation has low variability and relative semi-stability, but is closely related to cell process activity. The DNA methylation status of the CpG island of the promoter can regulate the expression of tumor-related genes and plays a key role in the occurrence and development of cancer (8). Moreover, the high plasticity of DNA methylation allows tumor cells to rapidly adapt to metabolic constraints or physiological changes during tumorigenesis (9). Therefore, the combination of transcriptome and methylation status can help identify novel markers, improve cancer diagnosis, and predict clinical outcomes.

β-Parvin (PARVB) is a member of the parvin protein family. The protein localizes to focal adhesions and plays important roles in cell adhesion, proliferation, and migration by activating multiple signaling pathways (10). Overexpression of PARVB has been associated with poor prognosis in human colorectal cancer (11) and tongue squamous cell carcinoma (12). PARVB has also been associated with epithelial-to-mesenchymal transition (EMT), a biological process in which epithelial cells undergo multiple biochemical changes, ultimately switching to a mesenchymal phenotype. Cells undergoing EMT lose their apical basolateral polarization and acquire a fibroblast-like morphology, characterized by weak cell adhesion and enhanced ability of the cell to migrate and spread (13, 14). The Janus Kinase/Signal Transducer and Activator of Transcription 3 (JAK/STAT3) signaling pathway is important in various types of cancer. Activation of this pathway leads to increased tumorigenic and metastatic capacity by enhancing EMT (15).

In this study, based on the Wilcoxon rank test, genes with significantly different expression in gliomas were selected from the transcriptome information in The Cancer Genome Atlas (TCGA) and Genotype-Tissue Expression (GTEx) databases. An integrative approach was used to identify GBM-related methylation-driven genes by combining the transcriptome and DNA methylation profiles from the TCGA database. A Cox survival predictive model-based risk score of six DNA-drive methylation genes (DMDGs) was successfully constructed. The score was effective in determining GBM patients with poor prognosis and displayed stronger predictive power in low-grade glioma (LGG) patients. The verification that EMT induced by PARVB can enhance the proliferation, migration, and invasion of GBM cells through the JAK2/STAT3 pathway emphasizes the value of the *PARVB* gene as a potential therapeutic target. Our findings provide new insights into the molecular mechanisms of GBM and prompt a more individualized therapy for this prevalent disease.

## Materials and Methods

### Sample Acquisition and Data Processing

RNA-sequence (RNA-seq) transcriptional data of normal tissues were obtained from the GTEx database (https://gtexportal.org/). RNA-seq transcriptome data, DNA methylation data, and clinicopathological information of gliomas were downloaded from TCGA database (https://gdc.cancer.gov/) and the Chinese Glioma Genome Atlas (CGGA) database (https://cgga.org.cn/).

### Selection of Differentially Expressed Genes in Glioma

RNA-seq transcriptome data obtained from normal tissues in GTEx and gliomas in TCGA were standardized using the limma package. The cutoff criteria were set as | log2 fold change (FC) | > 0.5 and *p* < 0.05. DEGs between normal and glioma tissues were selected based on the Wilcoxon rank test.

### Selection of DMDGs

The cutoff criteria of the DEGs and aberrantly methylated genes (AMGs) were set as | log2 FC | > 0.2 and *p* < 0.05 between LGG (selected astrocytoma only) and GBM samples. Combining the data of DEGs and AMGs in GBM, the MethylMix R package was used to screen DMDGs (16). The correlation coefficient was set to Cor < −0.4. DMDGs meeting the cutoff criteria were screened. The expression and methylation of these DMDGs were visualized based on the heatmap R package.

### Functional and Pathway Enrichment Analyses of DMDGs

Gene ontology (GO) analysis of DMDGs was performed using the Database of Annotation, Visualization and Integrated Discovery (DAVID) (https://david.abcc.ncifcrf.gov/). Kyoto Encyclopedia of Genes and Genomes (KEGG) pathway enrichment analysis was performed using KOBAS 3.0 (http://kobas.cbi.pku.edu.cn/). The GOplot R package was used to visualize the significantly enriched GO terms and KEGG pathways. Additionally, KEGG pathway analysis of PARVB was performed using Gene Set Enrichment Analysis (GSEA) software (www.gsea-msigdb.org). The cutoff criterion was set at p < 0.05.

### Construction, Evaluation, and Validation of DMDG Prognostic Signature

Univariable Cox regression analysis was utilized to select prognosis-related DMDGs (threshold value of genes was set as p < 0.05) in GBM patients. Applying multivariate Cox regression analysis optimized the constructed model by calculating the optimal AIC value. The smaller the AIC, the better the model, and the one with the smallest AIC is usually chosen. AIC=(2k-2L)/n. Genes with high correlation were removed in this process, and finally genes with large differences were selected to construct the model. Subsequently, the DMDG prognostic signature was constructed by the linear combination of the expression levels of DMDGs using the β coefficient calculated from multivariate Cox regression as the weight. The risk score for each patient was calculated as A (expression level of gene) *(beta1) + B*(beta2) + C*(beta3) +……. + (N-2)*(beta(n-2))+ (N-1)*(beta(n-1))+ (N)*(beta(n)). By setting the median value of the risk score as the cutoff value, GBM patients were separated into high-risk and low-risk groups. The difference in overall survival (OS) between the two groups was evaluated by Kaplan-Meier survival analysis using the log-rank test. Receiver operating characteristic (ROC) curves based on the survivalROC package were applied to assess the predictive performance of the prognostic signature of DMDGs within 1, 2, and 3 years. The predictive accuracy of the DMDG prognostic signature was evaluated using Harrell’s C-index with the survcomp package. The constructed risk model was re-scored by four previously published methylation-based gene signatures. The signature derived in this study was compared with these four other signature ROC curves. In addition to univariate Cox regression analysis, multivariate Cox regression analysis was performed to evaluate the resolution of the DMDG prognostic signature. Finally, the DMDG prognostic signature constructed using the TCGA database was validated in the CGGA database.

### Combined Survival Analysis Based on Expression and Methylation of DMDGs

Using the median values of gene expression and DNA methylation levels of DMDGs, patients were divided into low expression patients with hypermethylation and high expression patients with hypomethylation. The survival differences between the two groups were evaluated using Kaplan-Meier survival analysis.

### Cell Lines and Cell Culture

The LN229 human GBM cell line and HG7 patient-derived GBM cells were cultured as previously described (17). Cell line authentication was performed using short tandem repeat analysis (Genetic Testing Biotechnology, Jiangsu, China). Cell lines were actively passaged for up to 6 months. Only cells below passage 15 were used for the experiments.

### Chemical Reagents, Antibodies, and Transfection

The JAK2/STAT3 inhibitor WP1066 was purchased from Selleck Chemicals (Boston, MA, USA). PARVB overexpression plasmids and short hairpin RNA (shRNA) were purchased from GeneChem (Shanghai, China). Ara-C (cytarabine) was purchased from Sigma-Aldrich (St Louis, MO). Cells were transiently transfected with overexpression plasmids or shRNA for 2 weeks of puromycin selection to obtain stable cells. Forty-eight hours after transfection, western blot analysis of the collected cells was performed.

### Immunoblotting and Immunohistochemistry

GBM samples or cells were harvested using RIPA supplied with proteinase and phosphatase inhibitor cocktails (Selleck Chemicals, Shanghai, China). The protein concentration was determined using a BCA Assay Kit. Western blot assays were performed as described previously (18). The antibodies used are listed in **Supplementary Table S1**.

Paraffin-embedded GBM tissues were obtained from patients treated at First Affiliated Hospital of Nanchang University who provided informed consent. The study was approved by the hospital’s institutional ethics committee. Immunohistochemistry was performed as described previously (19). Clinical GBM samples were immunostained with primary antibodies against PARVB, E-cadherin, Claudin-1, N-cadherin, and vimentin at 4°C overnight. After washing in PBS, the tissues were exposed to secondary antibody (horseradish peroxidase [HRP]-conjugated anti-mouse/rabbit polymeric antibody) for 30 min. The 3,3’diaminobenzidine (DAB) Staining Kit (Zsgb Bio Inc., Beijing, China) was used as a chromogen for 1 min of incubation to allow for proper brown color development.

### Cell Proliferation Assay

Cell Counting Kit 8 (Dojindo, Kumamoto, Japan) was used to detect cell proliferation. The fluorescence at 450 nm was recorded using a microplate reader.

### Colony Formation Assay

After pancreatin digestion of logarithmic growth phase cells, the cells were harvested and resuspended in complete medium [basal medium + 10% fetal bovine serum (FBS)]and counted. Cells were seeded at 800 cells/well in 6-well plate culture plates for each experimental group. Cells were cultured for 8 days. One milliliter of Crystal violet dye solution was added to each well and the cells were stained for 30 min. Cells were washed several times with PBS, the number of colonies was captured using an Olympus camera (Tokyo, Japan) and counted using ImageJ software.

### Three-Dimensional (3D) Spheroid Cell Invasion Assay and Transwell Migration Assay

The 3D spheroid cell invasion assay was performed using a Cultrex^®^ 96-well 3D Spheroid BME Cell Invasion Assay kit (Trevigen, Gaithersburg, MD, USA). Approximately 3×10^3^ cells were resuspended in 50 µL of 1× spheroid formation ECM and were added to each well of a 96-well plate (Helgerman CT, Gaithersburg, MD, 20877). The plate was centrifuged at 200 ×g for 5 min, and incubated at 37°C for 72 h to promote the formation of cell spheres. Next, 50 μL of the invasion matrix/well plate was placed on ice for 10 min, followed by centrifugation at 300×g for 5 min at 4°C. The plates were then incubated at 37°C for 3 days. The cells were imaged using an Axio Imager M2 microscope (Carl Zeiss, Jena, Germany).

Transwell assays were performed in 24-well cell culture chambers with 8 mm pore Transwell inserts precoated with Matrigel. In brief, cells were seeded in 200 μL of culture medium containing 1% FBS. Five hundred microliters of medium containing 50% FBS was added to each lower chamber. After 24 h, the cells were fixed and stained with hematoxylin and eosin. Cells passing through the membrane were imaged (10×) and randomly counted into five independent fields.

### Statistical Analyses

Statistical analyses were conducted using the R statistical package (R version 4.0.2). A two-tailed p < 0.05 was considered a significant difference between groups. Significant differences between the groups were estimated using the Student’s t-test. One-way analysis of variance (one-way ANOVA) was performed for at least three groups.

## Results

### Selection of Significant DEGs in Glioma

**Figure 1:**
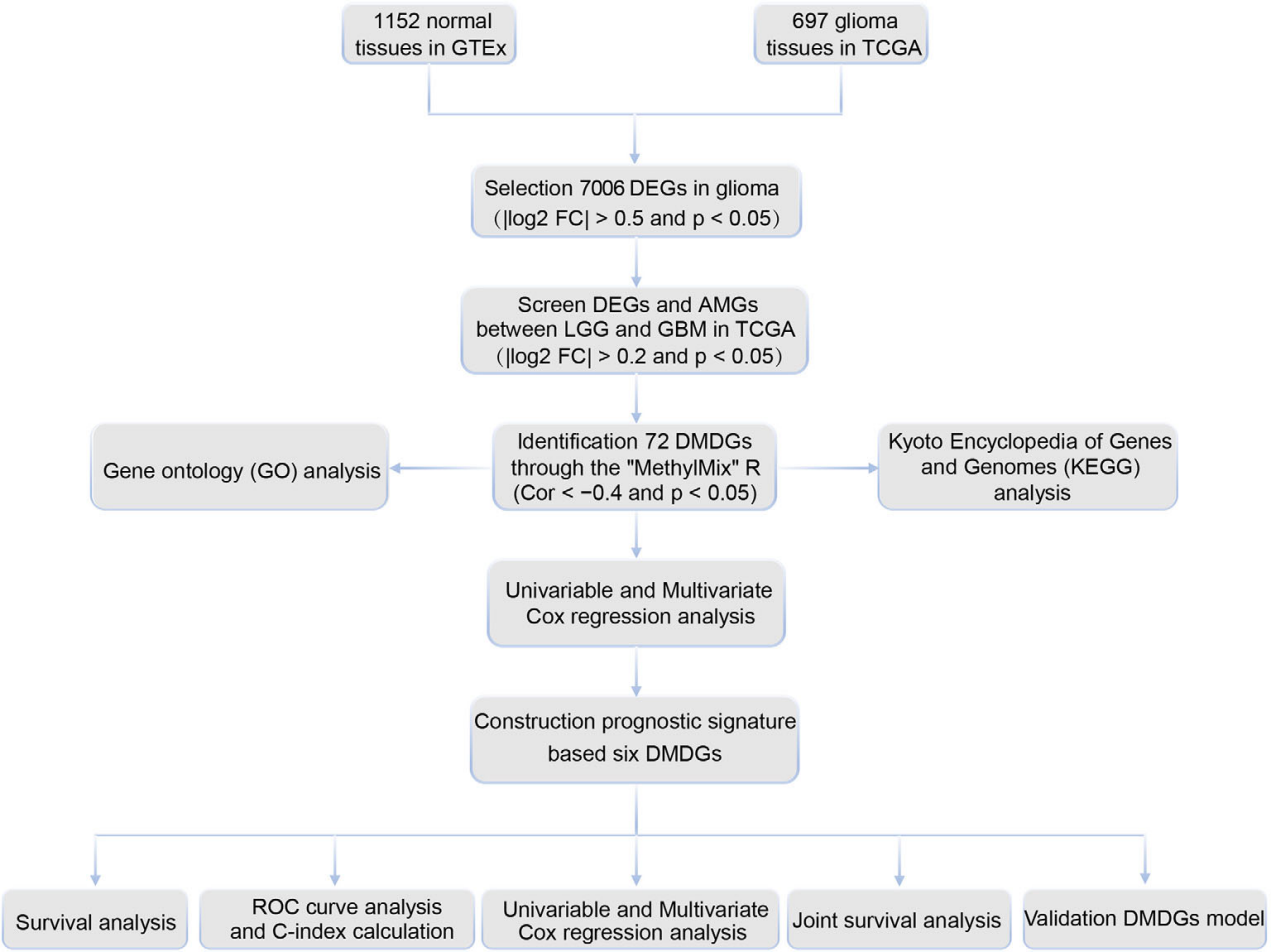
Flow chart of the exploration of DMDGs in GBM.

The cutoff criteria were set as | log2 FC | > 0.5 and p < 0.05. Based on RNA-seq data of 1152 normal tissues in GTEx and 697 gliomas (529 LGG and 168 GBM) in TCGA, 7006 DEGs were selected based on the Wilcoxon rank test between normal and glioma tissues. Of these, 3137 genes were upregulated and 3845 genes were downregulated (**Supplementary Table S2**). A filtering flow chart for the DMDGs is shown in **Figure 1**.

### Identification of DMDGs in GBM

**Figure 2:**
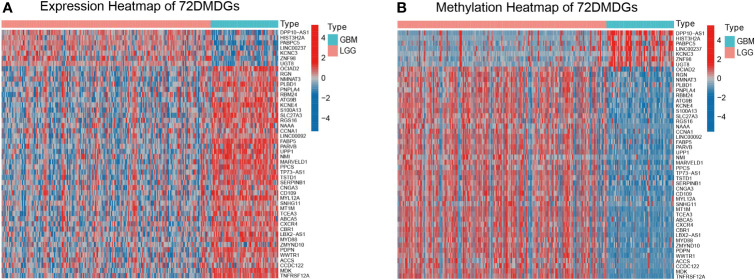
Expression and methylation heatmap of 72 DMDGs in glioma. **(A)** Expression patterns of 72 DMDGs. Colors from blue to red denote the trend from downregulated to upregulated genes between LGG and GBM tissues. **(B)** Methylation patterns of 72 DMDGs. Colors from blue to red denote the trend from hypermethylation to hypomethylation between LGG and GBM tissues. DMDGs, DNA methylation-driven genes; LGG, low grade glioma; GBM, glioblastoma multiforme.

The 7006 DEGs selected between normal and glioma tissues (**Supplementary Table S2**) were analyzed by the Wilcoxon rank test to further screen for DEGs and AMGs in 195 LGG (astrocytoma only) and 63 GBM (complete transcriptome and DNA methylation profiles) samples in TCGA. In the MethylMix R package, the screening criteria of DEGs and AMGs was set as | log2FC | > 0.2, p < 0.05 and Cor < −0.4. Seventy-two DMDGs were identified, of which nine were hypermethylated and 63 were hypomethylated (**Supplementary Table S3**). The expression level and methylation value of DMDGs are shown in a heat map (**Figure 2A, B** and **Supplementary Figure S1A**).

### Functional Enrichment Analysis of DMDGs in GBM

**Figure 3:**
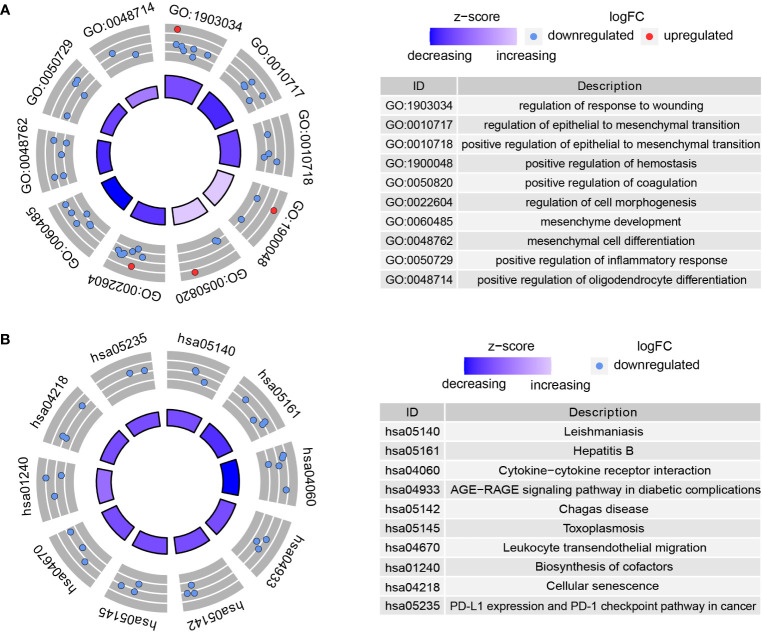
Functional enrichment analysis of DMDGs in GBM. **(A)** GO enrichment analysis. **(B)** KEGG pathway enrichment analysis. The color of inner circle represents z scores: red dots indicate high z scores while blue dots indicate low z scores. The band thickness of inner circle represents the significance of GO terms. The outer circle represents the expression level of DMDGs in each enriched GO term: the dot colors from blue to red denote the trend from hypermethylated to hypomethylated genes.

The possible functions and pathways of the 72 DMDGs in GBM were explored using GO functional enrichment analysis and KEGG pathway enrichment analysis. Among biological processes (BP), molecular function (MF), and cellular component (CC), functional analysis showed that DMDGs were enriched in positive regulation of EMT, regulation of cell morphogenesis, mesenchyme development, mesenchymal cell differentiation, positive regulation of inflammatory response, and positive regulation of oligodendrocyte differentiation (**Figure 3A**). In addition, KEGG pathway enrichment analysis showed that the DMDGs were enriched in programmed cell death ligand 1 expression and programmed cell death protein 1(PD-1) checkpoint pathway in cancer, cellular senescence, and advanced glycation end product (AGE)-receptor for AGE (RAGE) signaling pathway in diabetic complication phenotype-related pathways (**Figure 3B**).

### Construction of DMDG Prognostic Model

**Figure 4:**
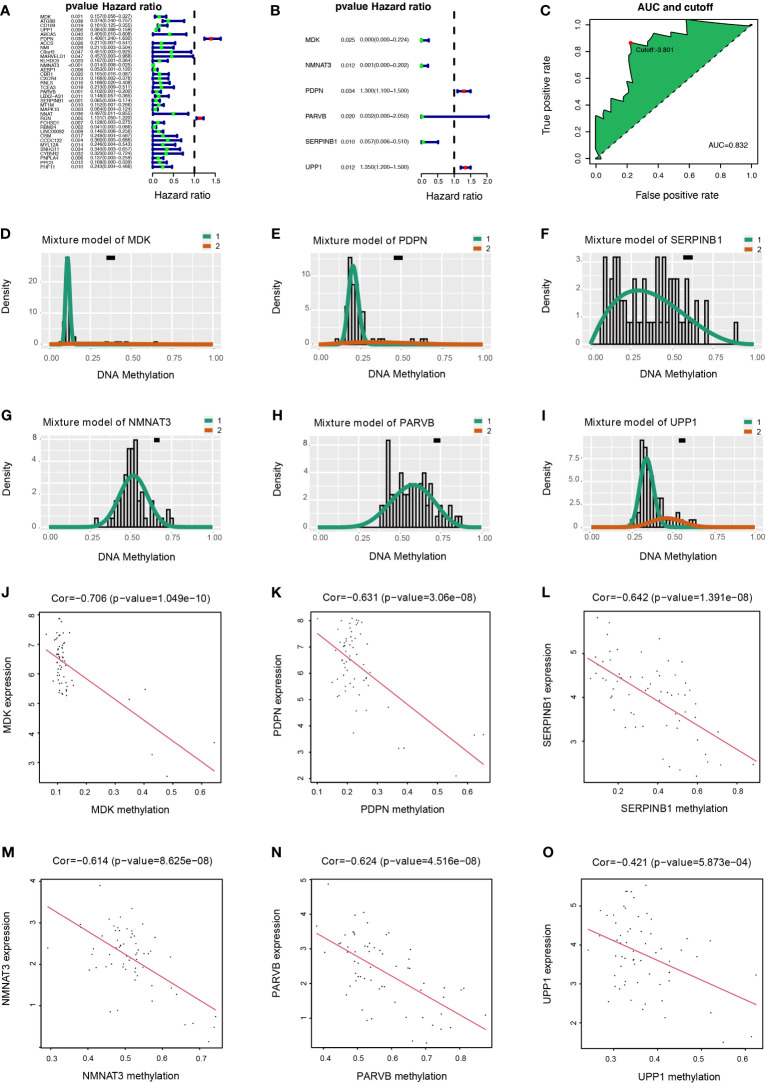
Construction of DMDG prognostic model and Relationship of expression levels and methylation values of six DMDGs. **(A)** Forest plot of 35 DMDGs selected by univariate Cox regression analysis associated with GBM survival in the training set. **(B)** Forest plot of 6 DMDGs selected by multivariate Cox regression analysis associated with GBM survival and construction risk model. **(C)** The AUC value and cut-off point obtained in the risk model. **(D-I)** Distribution map of the methylation level of the six genes (*MDK*, *NMNAT3*, *PDPN*, *PARVB*, *SERPINB1*, and *UPP1*) comprising the risk model. The X- and Y-axes represent the degree of methylation and the number of methylated samples, respectively. The black horizontal line indicates the distribution of methylation degree in normal samples. **(J–O)** Correlation of expression levels and methylation values of the *MDK*, *NMNAT3*, *PDPN*, *PARVB*, *SERPINB1*, and *UPP1* genes in the risk model. The X- and Y-axes indicate the degree of methylation and the level of gene expression, respectively.

Univariate Cox regression analysis was used to determine the prognostic value of 72 DMDGs. Thirty-five genes were associated with prognosis (**Figure 4A** and **Supplementary Table S4**). Subsequently, multivariate Cox regression analysis was used to construct the DMDG prognostic signature based on six DMDGs (**Figure 4B**): midkine (MDK), nicotinamide/nicotinic acid mononucleotide adenylyltransferase 3 (NMNAT3), podoplatin (PDPN), PARVB, leukocyte elastase inhibitor (SERPINB1), and uridine phosphorylase 1 (UPP1). The coefficient of each gene was calculated by multivariate Cox regression analysis (**Table 1**). The risk score for each patient was calculated using the following formula: the DMDG prognostic model risk score formula was MDK (gene expression level) *(-11.945) + NMNAT3*(-7.145) + PDPN*(9.446) + PARVB*(-3.456) + SERPINB1*(-2.858) + UPP1*(6.083). The 1 year’ AUC value is 0.832 and cut-off point is -3.801 in the risk model (**Figure 4C**). The methylation degree of six DMDGs in LGG and GBM tissues are shown in **Figure 4D–I**. The methylation levels of the *MDK*, *NMNAT3*, *PDPN*, *PARVB*, *SERPINB1*, and *UPP1* genes were inversely proportional to their expression levels (**Figure 4J–O**).

**Table 1:** Coefficients based on a multivariate Cox regression analysis of the selected genes.

Gene	Coef	HR	HR.95L	HR.95H	p-value
MDK	-11.945	6.49E-06	1.88E-10	0.224	0.025
NMNAT3	-7.145	7.89E-04	3.08E-06	0.202	0.012
PDPN	9.446	1.323	1.1243	1.5684	0.074
PARVB	-3.456	0.032	4.86E-04	2.05	0.105
SERPINB1	-2.858	0.057	0.006	0.51	0.01
UPP1	6.083	1.3585	1.219	1.584	0.017

HR, hazard ratio.

### DMDG Prognostic Model Is an Independent Prognostic Factor

**Figure 5:**
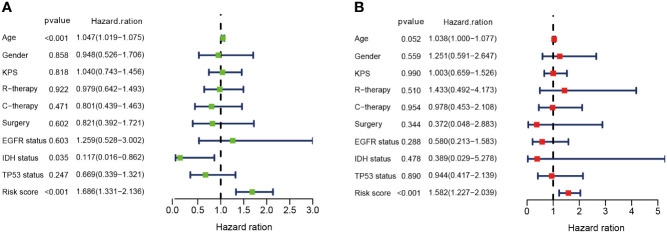
DMDG prognostic model is an independent prognostic factor. **(A)** Univariate analysis of the risk score and other clinical information in TCGA GBM cohort. **(B)** Multivariate analysis of the risk score and other clinical information in TCGA GBM cohort. KPS, Karnofsky Performance Status; R-therapy, radiotherapy; C-therapy, chemotherapy; EGFR, Epidermal Growth Factor Receptor; IDH, isocitrate dehydrogenase.

In univariate Cox analysis, age (p < 0.001, hazard ratio [HR] =1.047, 95% confidence interval [CI] = 1.019-1.075) and risk score (p < 0.001, HR = 1.686, 95% CI = 1.331-2.136) were significantly correlated with OS (**Figure 5A**). In multivariate Cox analysis, only risk score (p < 0.001, HR = 1.582, 95% CI = 1.227-2.039) was an independent prognostic factor (**Figure 5B**).

### Evaluation and Comparison of DMDG Prognostic Model

**Figure 6:**
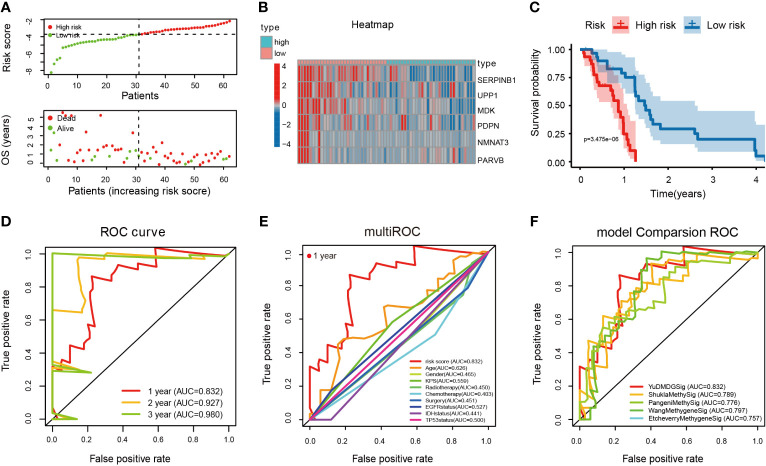
Evaluation and comparison of DMDG prognostic model. **(A)** Risk score and survival status analysis of DMDG prognostic signature: the green dots represent low-risk patients and red dots represent high-risk patients. **(B)** The expression pattern of DMDG prognostic signature in the low- and high-risk groups. **(C)** Survival analysis of the two subgroups in DMDG prognostic signature. **(D)** ROC curve analysis for assessing the predictive power of the risk model within 1-, 2-, and 3-years. **(E)** Multivariate ROC curve analysis showing that the superior prognostic performance of the DMDG prognostic model compared to other clinical information within 1 year. **(F)** AUCs of the ROCs for our and the four other gene signatures within 1 year. The results demonstrate that the risk signature we constructed exhibits the most excellent predictive power.

According to the median DMDG risk score, GBM patients with complete clinical data were divided into high-risk and low-risk cohorts. The distribution of risk scores for the cohorts is shown in **Figure 6A**. As the risk score increased, the prevalence of death increased and the number of surviving patients decreased. The methylation patterns of the six DMDGs in GBM are shown in **Figure 6B**. Survival analysis showed that the low-risk cohort had longer OS than the high-risk cohort (**Figure 6C**). ROC curve analysis showed that the area under the curve (AUC) values of the ROC curve were 0.832, 0.927, and 0.980 within 1, 2, and 3 years, respectively (**Figure 6D**). The C-index values (index = 0.704, se = 0.038, lower = 0.629, upper = 0.779, p < 0.001) further demonstrated the superior predictive power of the DMDG prognostic signature. Compared with other clinical information (including age, sex, Karnofsky performance scale, radiotherapy, chemotherapy, surgery, epidermal growth factor receptor status, isocitrate dehydrogenase status, and tumor protein 53 status), multivariate ROC curve analysis indicated that the risk model had an excellent prediction efficiency (**Figure 6E**). We compared our signature with four previously published GBM methylation-based gene signature panels: the 9-gene methylation signature (20), 16-gene methylation signature (21), 8-gene methylation signature (22), and 13-gene methylation signature (23). The ROC curves of the different models were visualized using survivalROC packages. Our signature was superior to the 9-, 16-, 8-, and 13-gene methylation-based gene signatures (AUCs of 0.832, 0.789, 0.776, 0.796, and 0.757, respectively) (**Figure 6F**). Shukla et al. (20) and Pangeni et al. (21) considered methylation status alone. Although Wang et al. (22) and Etcheverry et al. (23) jointly analyzed gene expression levels and methylation values, no correlation coefficients were introduced. These reasons make the four methylation signatures inferior in predictive ability to our model. Together, these data illustrate the excellent identification of high-risk patients using our model.

### Validation of DMDG Prognostic Model

**Figure 7:**
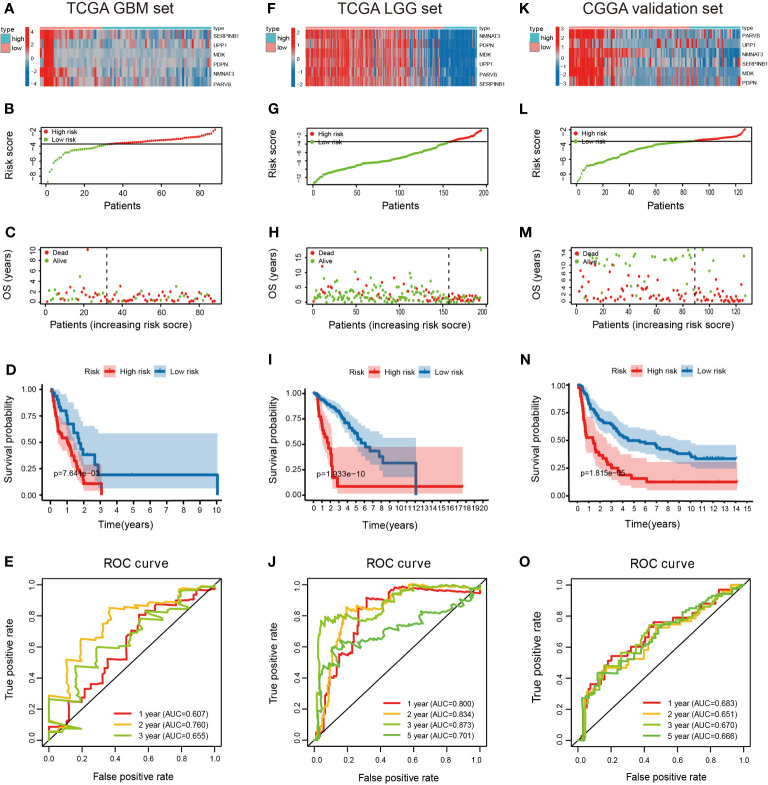
Validation of the DMDG based risk score model in TCGA training cohort and CGGA validation cohort. **(A)** Heatmap of the 6-gene expression pattern: red represents upregulated genes and blue represents downregulated genes between low-risk and high-risk groups, **(B)** risk score analysis, **(C)** survival status analysis and **(D)** survival analysis in low- and high-risk groups in TCGA GBM set: the blue dots represent low-risk patients and red dots represent high-risk patients. **(E)** ROC curve analysis in TCGA GBM set within 1-, 2-, and 3-years. **(F–I)** Heatmap of the 6-gene expression pattern, risk score analysis, survival status analysis, and survival analysis in low- and high-risk groups in TCGA LGG set. **(J)** ROC curve analysis in TCGA LGG set within 1-, 2-, 3- and 5-years. **(K–N)** Heatmap of the 6-gene expression pattern, risk score analysis, survival status analysis and survival analysis in low and high-risk groups in CGGA validation set. **(O)** ROC curve analysis in CGGA validation set within 1-, 2-, 3- and 5-years.

The predictive ability of the 6-gene model was validated in a GBM patient TCGA cohort (n = 88) who were not involved in the construction of the model. Based on the cutoff value (-3.22), 56 patients were assigned to the high-risk group and 32 patients to the low-risk group. The expression pattern of these six DMDG prognostic signatures in the low- and high-risk groups are shown in **Figure 7A**. The distributions of the risk scores are shown in **Figure 7B**. Moreover, the distributions of risk scores and OS status of each patient in **Figure 7C** indicated good discrimination between the low-risk and high-risk groups. Survival curves demonstrated that high-risk patients had a significantly poorer prognosis than low-risk patients (p < 0.01) (**Figure 7D**). The AUCs of the ROC curves were 0.607, 0.760, and 0.655 within 1, 2, and 3 years, respectively (**Figure 7E**). We also validated the prognostic value of the risk signature in an LGG patient TCGA cohort (n = 529). Based on the cutoff value (-3.22), LGG patients with complete clinical data were divided into high-risk (n = 71) and low-risk cohorts (n = 458). The expression pattern of the prognostic signature, risk scores, OS status, and survival analysis (p < 0.001) are shown in **Supplementary Figures S2A–D**). The AUCs of the ROC curves were 0.830, 0.774, 0.748, and 0.680 within 1, 2, 3, and 5 years, respectively (**Supplementary Figure S2E**). In addition, the prognostic power of the risk model was validated in an LGG (astrocytoma only) patient TCGA cohort (n = 195). Based on the same cutoff value, a total of 37 patients were categorized into the high-risk group, while the remaining 158 patients were classified into the low-risk group. The expression pattern of DMDG prognostic signature, risk scores, and OS status are shown in **Figure 7F–H**. The low-risk column had a more optimized OS than the high-risk column (p < 0.001) (**Figure 7I**). The AUCs of the ROC curves were 0.800, 0.834, 0.873, and 0.701 within 1, 2, 3, and 5 years, respectively, demonstrating the excellent predictive ability of the risk score (**Figure 7J**). In addition to TCGA database, glioma patients (n = 127) available in the CGGA database were also used to validate our risk model (**Figure 7K–O**). The main feature of the present study is that grouping occurred between LGG and GBM, but not between normal and GBM. The study limited the singularity of tumor types, in which all tumors chose astrocytoma (selection astrocytoma only in LGG, while GBM was a high-grade astrocytoma). Therefore, our risk model was more suitable for LGG (especially astrocytoma) and had a higher prediction accuracy.

### Overexpression of PARVB Is Correlated With Shorter Survival and Upregulated PARVB-Induced EMT in GBM Patients

**Figure 8:**
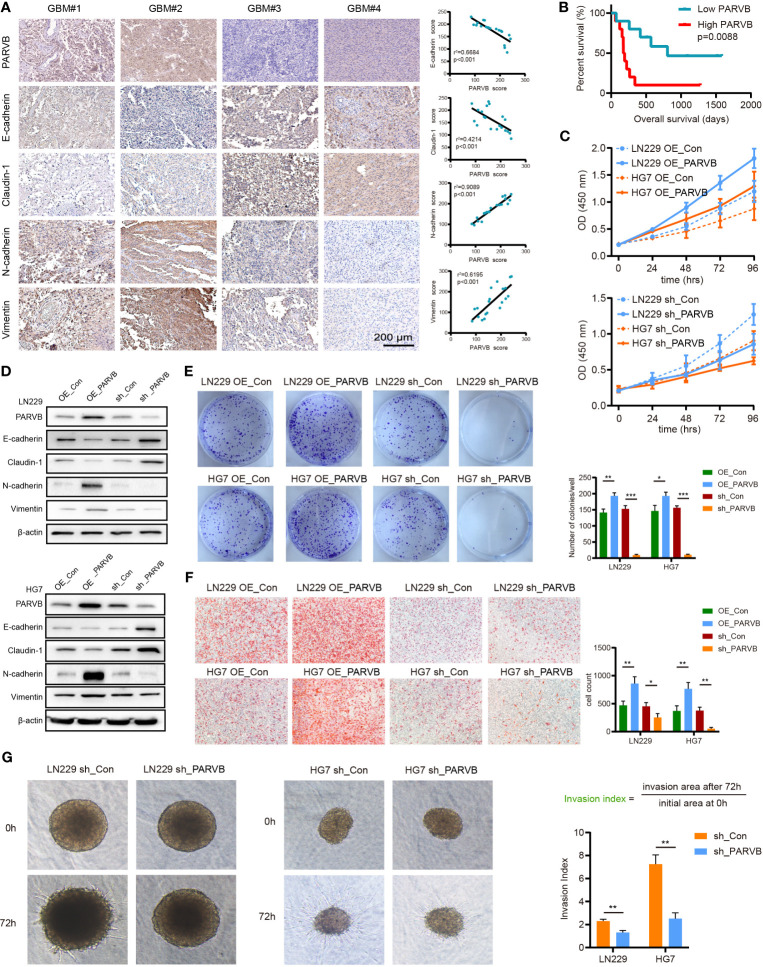
PARVB regulates proliferation, migration, invasion, and EMT *in vitro* of GBM cells. **(A)** Immunohistochemistry **(IHC)** analysis was performed on twenty GBMs, representative images of 4 GBMs (GBM#1 and GBM#2 high PARVB expression, GBM#3 and GBM#4 low PARVB expression) are shown. IHC of epithelial markers (E-cadherin and Claudin-1), mesenchymal markers (N-cadherin and Vimentin), and PARVB in tumor sections from GBM samples. Scale bar = 200 μm. PARVB score was positively correlated with the mesenchymal markers (N-cadherin and Vimentin) score and negatively correlated with the epithelial markers (E-cadherin and Claudin-1) score. **(B)** Kaplan-Meier analysis in low expression and high expression PARVB groups in 20 GBM patients: the green dots represent low expression PARVB patients and red dots represent high expression PARVB patients. **(C)** CCK-8 analysis show that overexpression of PARVB accelerates the proliferation and PARVB knockdown reduces the proliferation in LN229 and HG7 cells at the indicated times. **(D)** Western blot analysis of the indicated proteins in LN229 and HG7 GBM cells after PARVB overexpression or knockdown. Result shows that PARVB expression was positively correlated with mesenchymal marker (N-cadherin and Vimentin), and negatively correlated with epithelial marker (E-cadherin and Claudin-1) in LN229 and HG7 cells. **(E)** Colony formation assay shows that PARVB overexpression promotes the proliferation and PARVB knockdown reduces the growth of LN229 and HG7 GBM cells in a 6-well dish (800 cells per well) for 8 days (n = 3). Representative images of colonies are shown. The assays were determined from three independent experiments, quantification data are expressed as average ± SD. **(F, G)** Transwell migration analysis and 3D spheroid cell invasion assay showing the effect of PARVB overexpression and knockdown on the LN229 and HG7 GBM cells. Representative images and statistical analysis of three independent assays are shown, quantification data are expressed as average ± SD. Significant results are indicated as *p < 0.05, **p < 0.01, and ***p < 0.001.

We performed a single gene survival analysis for the prognostic model, dividing the genes into low methylation high expression group and high methylation low expression group, using the median of methylation value as the cutoff values. MDK, SERPINB1, NMNAT3, PARVB, and UPP1 had prognostic value. The low methylation, high expression group was associated with poor prognosis (p < 0.05; **Supplementary Figure S3A–E**). PARVB has a broad spectrum of biological actions during cancer development (11, 12), but its mechanistic exploration in GBM has been less studied and was therefore chosen for further study. Immunohistochemical analysis revealed that PARVB expression was positively correlated with the mesenchymal markers N-cadherin and Vimentin, and negatively correlated with the epithelial markers E-cadherin and Claudin-1 in GBM tissues (**Figure 8A**). Survival curves demonstrated that high expression PARVB patients had a significantly poorer prognosis than low expression PARVB patients in 20 GBM patients (p = 0.0088) (**Figure 8B**). Western blot analysis showed that PARVB overexpression led to the downregulation of epithelial markers and upregulation of mesenchymal markers, whereas PARVB knockdown resulted in the opposite effect (**Figure 8D**). CCK8, colony formation, Transwell, and 3D invasion assays showed that PARVB overexpression promoted proliferation, migration, and invasion in LN229 and HG7 cells. In contrast, PARVB knockdown significantly inhibited proliferation, migration, and invasion of LN229 and HG7 cells (**Figures 8C, E–G**). In addition, we observed same effects in the results of migration and invasion even though treated with Ara-C (cytarabine, 3μg/mL), which was usually used to inhibit cell proliferation. (**Supplementary Figures S3F, G**). These data suggest that PARVB promotes malignant behavior by regulating EMT in GBM.

### PARVB Promotes EMT by Activating the JAK-STAT Pathway

**Figure 9:**
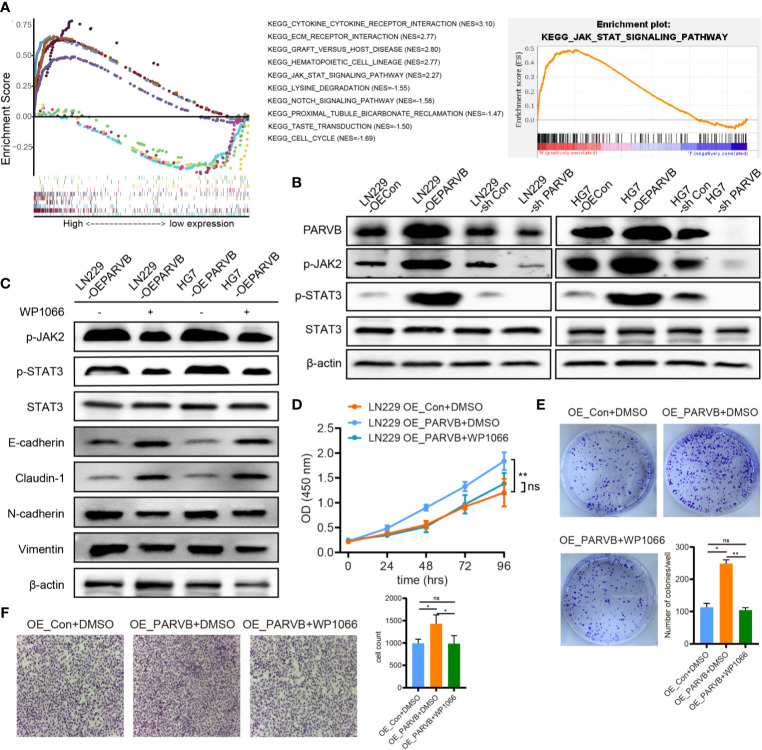
PARVB promotes EMT by activating the JAK2/STAT3 pathway. **(A)** GSEA of the top five negative and positive function gene sets associated with PARVB expression. The high expression PARVB group was positively correlated with the JAK-STAT pathway. **(B)** Western blot analysis shows that p-JAK2 and p-STAT3 expression was upregulated by PARVB overexpression and was downregulated by PARVB knockdown in LN229 and HG7 cells. **(C)** Western blot analysis of the indicated proteins in LN229 and HG7 GBM cells overexpressing PARVB with WP1066 (200 nM) treatment. Activating JAK2/STAT3 pathway significantly promoted EMT in LN229 and HG7 cells. Conversely, inhibitor of JAK2/STAT3 signaling partially rescued EMT in LN229 cells. **(D)** CCK-8 assay analysis of the effect of WP1066 on LN229 cells after overexpression of PARVB at the indicated times. Silencing JAK2/STAT3 signaling *via* WP1066 partly inhibited the increased proliferation in PARVB-overexpressing LN229 cells, whereas activating JAK/STAT signaling promotes proliferation of PARVB-knockdown LN229 cells. **(E)** Colony formation assay of the effect of WP1066 on the growth of LN229 GBM cells after overexpression of PARVB in a 6-well dish (800 cells per well) for 8 days. Representative images and statistical analysis of three independent assays are shown, quantification data are expressed as average ± SD. **(F)** Transwell migration analysis showing the effect of WP1066 on LN229 cells after overexpression of PARVB on LN229 GBM cells. Silencing JAK2/STAT3 signaling *via* WP1066 partly inhibited the increased migration and invasion in PARVB-overexpressing LN229 cells, whereas activating JAK/STAT signaling partially rescued the inhibited migration and invasion of PARVB-knockdown LN229 cells. Representative images and statistical analysis of three independent assays are shown, quantification data are expressed as average ± SD. Significant results are presented as ns (non-significant), *p < 0.05, and **p < 0.01.

To further investigate the mechanism underlying the promotion of EMT by PARVB, GSEA of single genes was performed. The analysis demonstrated that the high PARVB expression group was significantly enriched in the JAK-STAT signaling pathway (p < 0.001; **Figure 9A**). Knockdown of PARVB decreased phosphorylated (p)-JAK2 and p-STAT3 levels, and PARVB overexpression increased p-JAK2 and p-STAT3 levels (**Figure 9B**). JAK2 inhibitor prevented PARVB-induced EMT in LN229 and HG7 cells (**Figure 9C**). To clarify whether the promotion of EMT by PARVB involved the JAK2/STAT3 pathway, the biological effects of the JAK2/STAT3 pathway were assessed in PARVB-overexpressing GBM cells. Inhibition of JAK2/STAT3 signaling prevented PARVB-induced proliferation, migration, and invasion phenotypes in GBM cells overexpressing PARVB (**Figure 9D–F**). We also observed similar results of migration and invasion even though treated with Ara-C (**Supplementary Figure S4A**). These data demonstrate that PARVB promotes EMT through the JAK2/STAT3 pathway.

## Discussion

Glioma is the most common malignant brain tumor, and its prognosis is not ideal. GBM is highly malignant with high mortality (24). Even with standard treatment, surgical resection with the maximum safe range in combination with postoperative concurrent radiotherapy and TMZ chemotherapy, the OS of patients with GBM remains unsatisfactory (25). Parsons et al. (26) found that IDH1 mutations occurred in a large number of young and secondary GBMs, and for the first time highlighted the potential of the mutant gene in the classification of GBM subtypes. A large number of in-depth studies on the biological behavior and molecular markers of GBM have improved our understanding of the pathogenesis of GBM. In 2016, the revised classification of central nervous system tumors was constructed based on genetic and epigenetic alterations, including IDH 1/2, MGMT methylation, 1p/19q codeletion, and EGFR (27). DNA methylation alterations, as a type of epigenetic mechanism, regulate gene expression levels *via* methylation status and are important in tumorigenesis and progression of GBMs (28, 29). During the progression and recurrence of gliomas, DNA methylation is lost (30). The low methylation status leads to the increased expression of related oncogenes. GBM patients with MGMT promoter methylation are more sensitive to chemoradiotherapy and have excellent survival benefits (5). Klughamer et al. demonstrated that aberrant DNA methylation is associated with patient prognosis and can serve as a therapeutic target in GBM (31). In addition, considering the complexity and heterogeneity of GBMs, combined molecular markers are better than single biomarkers in the prognosis of glioma. Thus, DMDGs can be used as a tool for early diagnosis, risk stratification, and prognosis prediction, and can be therapeutic targets in GBM.

Traditional DMDG screening occurs in both normal and tumor groups. In this study, screening events were conducted between low-grade astrocytomas (only astrocytomas were selected in LGG) and high-grade astrocytomas (GBM). Based on RNA-seq data of normal tissues in the GTEx database and gliomas in TCGA, 7006 significant DEGs were first selected between normal and glioma tissues. In the TCGA database, the 7006 significant DEGs were utilized to further screen for DEGs and AMGs in LGG and GBM. Seventy-two DMDGs were identified in GBM using the MethylMix algorithm. Function and pathway analyses showed that these genes were mainly enriched in the BP group and were mainly involved in cancer-related pathways, including positive regulation of EMT, positive regulation of oligodendrocyte differentiation, and positive regulation of gliogenesis, suggesting that DMDGs play an important role in the malignant biological behavior of GBM. Subsequently, a novel DMDG (MDK, NMNAT3, PDPN, PARVB, SERPINB1, and UPP1) risk signature was constructed to act as a reliable predictor by univariate and multivariate Cox analyses. Based on the DMDG prognostic model risk score, patients with GBM in the TCGA database could be divided into high-risk and low-risk cohorts. Survival analysis showed that low-risk GBM had a significantly superior survival benefit for high-risk GBM. The AUCs of the ROC of the 6-gene signature were 0.832, 0.927, and 0.980 within 1, 2, and 3-years, respectively. The C-index value of 0.704 indicated superior prediction. In addition, the DMDG model based on the risk score was demonstrated to be an independent prognostic factor using univariate and multivariate Cox analyses. Importantly, our model showed a strong prognostic value in comparison with four other methylation-based gene signature groups. The AUCs of the ROCs of our and the 9-, 16-, 8-, and 13-gene signatures were 0.832, 0.789, 0.776, 0.796, and 0.757, respectively. Finally, the six risk signature genes were validated in the TCGA GBM cohort (n = 88), TCGA LGG cohort (n = 529), TCGA LGG (only astrocytoma) cohort (n = 195), and CGGA glioma cohort (n = 127). The AUC of the ROC curves indicated that the risk score had good prediction ability.

Joint survival analysis combining the methylation values and the expression levels of DMDGs indicated that genes with high expression levels (*MDK*, *SERPINB1*, *NMNAT33*, *PARVB*, and *UPP1*) were significantly associated with poor prognosis and had the potential to be therapeutic targets in GBM. Among the six DMDGs, MDK (32, 33), PDPN (34, 35) and SERPINB1 (36) levels have been shown to be significantly higher in glioma tissues than in normal tissues. Enhanced levels of these genes is associated with the deterioration of prognosis in patients with GBM (37–40). In addition, MDK (32, 38, 41, 42), PDPN (43, 44) and SERPINB1 (45) have been recognized as novel biomarkers and potent therapeutic targets for the treatment of GBM. In glioma, studies on NMNAT3, PARVB, and UPP1 are sparse. Only one bioinformatic analysis of UPP1 expression in gliomas has been published. It indicated that UPP1 expression is positively correlated with the grade of gliomas (46). Finally, because PARVB gene function in GBM has not yet been reported, it was further investigated. The mammalian parvin protein family includes three members (α-, β-, and γ-Parvin) that have key roles in actin reorganization and focal adhesions (47). β-Parvin (PARVB) is overexpressed in human colorectal cancer (11) and tongue squamous cell carcinoma (12), and is closely associated with tumor progression. Importantly, overexpression of nuclear β-catenin and downregulation of E-cadherin were observed in human colorectal cancer featuring highly expression of PARVB protein (11). In PARVB knockdown oral cancer cells, loss of fibroblast-like structures at the wound edge was found (12). These observations are consistent with the potential close association between PARVB overexpression and EMT. Such an association between PARVB and EMT has not been previously reported in patients with GBM. EMT driven by signaling pathways is a biological process in which a polarized epithelial cell sheet undergoes multiple biochemical changes, ultimately leading to a mesenchymal phenotype characterized by cells with weak cell adhesion and enhanced migration (48). Moreover, cells undergoing EMT lose their apical basolateral polarization and acquire a fibroblast-like morphology, which increases their ability to migrate, spread, and disseminate to surrounding tumor tissues or distant sites (13, 14, 49, 50). Function and pathway analyses showed that DMDGs (MDK, SERPINB1, NMNAT33, PARVB, and UPP1) were mainly involved in the positive regulation of EMT. Our study found that patients with higher PARVB tumor expression had significantly worse survival rates. The underlying mechanism by which PARVB promotes EMT was further investigated. GSEA of single genes demonstrated that highly expressed PARVB positively correlated with the JAK-STAT signaling pathway. Overexpression of PARVB *in vitro* can induce EMT, resulting in significantly increased cell proliferation, migration, and invasion. These findings imply that PARVB is involved in EMT-like processes in GBM. Silencing JAK2/STAT3 signaling partially inhibited the increased proliferation, migration, and invasion of PARVB-overexpressing cells *in vitro*. These data suggest that PARVB-induced EMT is at least partially mediated through the JAK2/STAT3 pathway.

Notably, this study differs in several ways from previous studies (20–23). First, the study introduced a large number of normal groups (GTEx database) to overcome the limitation of analysis due to the lack of normal groups in TCGA. Second, it simplified the types of tumor subtypes. Only astrocytoma patients were chosen as the screening samples. This made the analysis more precise and targeted. Third, the search for DMDGs selected LGG and GBM, rather than between normal and tumor groups, which could amplify the small and easily overlooked differences in gliomas in previous studies. Fourth, the introduction of a correlation coefficient (Cor < -0.4) in our model more convincingly resulted in strong predictive power. Fifth, our DMDG prognostic model is suitable for GBM as well as being more suitable and accurate for LGG. Sixth, this is the first study to report PARVB in GBM. PARVB induced EMT, and affected cell proliferation, migration, and invasion through the JAK2/STAT3 pathway.

## Conclusion

Combined analysis of transcriptome and DNA methylation profiles of TCGA was used to derive a 6-gene model-based risk score, screened from DMDGs. The approach allowed risk stratification survival prediction and personalized treatment plans for GBM. Our predictive model for DMDGs is better suited for LGGs than GBMs. Furthermore, we revealed that the *PARVB* gene can induce EMT to promote GBM cell malignant behavior partially through the JAK2/STAT3 pathway in GBM. Overall, we successfully constructed a prognostic model of DMDGS and validated the mechanism by which PARVB may be involved in regulating EMT in GBM.

## Data Availability Statement

The original contributions presented in the study are included in the article/**Supplementary Material**. Further inquiries can be directed to the corresponding authors.

## Ethics Statement

The studies involving human participants were reviewed and approved by Institutional Ethics Committee-approved study from the First Affiliated Hospital of Nanchang University. The patients/participants provided their written informed consent to participate in this study.

## Author Contributions

All authors contributed to the experimental design and the analysis of data in this study. Conception and design: PW, BH. Acquisition, analysis and interpretation of data: WY, LM and FW. Writing, review, and/or revision of the manuscript: WY. Administrative, technical, or material support: BH, PW. Study supervision: ZJ, BH. All authors contributed to the article and approved the submitted version.

## Funding

This work was supported by the Project of Nanchang Science and Technology Support Plan of Jiangxi Province, China (HONGKOZi[2021]129) from Wanli Yu, China Postdoctoral Science Foundation (2020M6703812020T130435) from by BH.

## Conflict of Interest

The authors declare that the research was conducted in the absence of any commercial or financial relationships that could be construed as a potential conflict of interest.

## Publisher’s Note

All claims expressed in this article are solely those of the authors and do not necessarily represent those of their affiliated organizations, or those of the publisher, the editors and the reviewers. Any product that may be evaluated in this article, or claim that may be made by its manufacturer, is not guaranteed or endorsed by the publisher.

## Supplementary Material

Supplementary Figure 1 Expression heatmap of 72 DMDGs in GBM.

Supplementary Figure 2 Validation of DMDGs prognostic model in TCGA LGG set (n=458).

Supplementary Figure 3 Joint survival analysis and Assessment of migration and invasion independent of proliferation.

Supplementary Figure 4 Assessment of migration independent of proliferation.

Supplementary Table 1:1 The antibody of western blot assays used in this study.

Supplementary Table 2:2 A total of 7006 DEGs were selected in GBM patients.

Supplementary Table 3:3 A total of 72 DMDGs were identified in GBM patients.

Supplementary Table 4:4 A total of 35 prognostically relevant DMDGs were used for prognostic model construction.

## Abbreviations

GBM, Glioblastoma; DMDGs, DNA methylation-driven genes; GTEx, Genotype-Tissue Expression; TCGA, The Cancer Genome Atlas; CGGA, Chinese Glioma Genome Atlas; ROC, Receiver operating characteristics; PARVB, β-Parvin; LGG, low grade glioma; EMT, epithelial-mesenchymal transition; IDH, isocitrate dehydrogenase; MGMT, O6-methylguanineDNA methyltransferase; TMA, temozolomide; FC, fold change; AMGs, aberrantly methylated genes; GO, Gene ontology; DAVID, Database of Annotation, Visualization and Integrated Discovery; KEGG, Kyoto Encyclopedia of Genes and Genomes; GSEA, Gene Set Enrichment Analysis; STR, short tandem repeat; shRNA, short hairpin RNA; BP, biological processes; MF, molecular function; CC, cellular component; KPS, Karnofsky Performance Status; EGFR, Epidermal Growth Factor Receptor; OS, over survival.
