# Supplementary material for: Construction of Novel Methylation-Driven Gene Model and Investigation of PARVB Function in Glioblastoma
Source: Front Oncol. 2021 Sep 10;11:705547. doi: 10.3389/fonc.2021.705547 (PMC8461318; doi:10.3389/fonc.2021.705547)
Supplement: Supplementary Figure 1 — Expression heatmap of 72 DMDGs in GBM. [file Presentation_1.pdf]

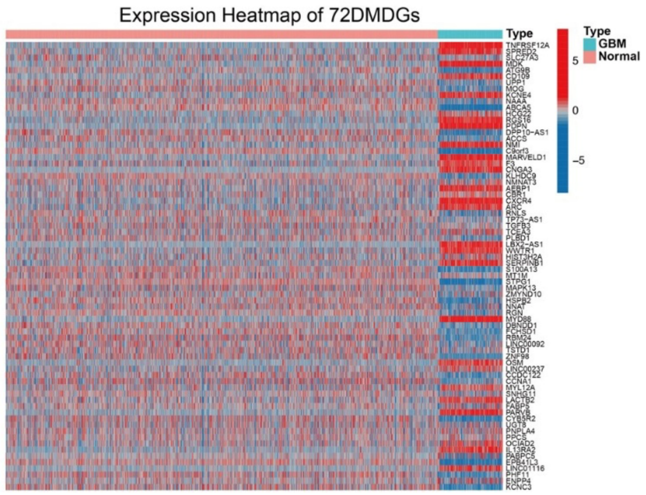

Figure S1 | Expression heatmap of 72 DMDGs in GBM

Expression patterns of 72 DMDGs. Red represents upregulated genes and blue represents downregulated genes between GBM and normal tissues.

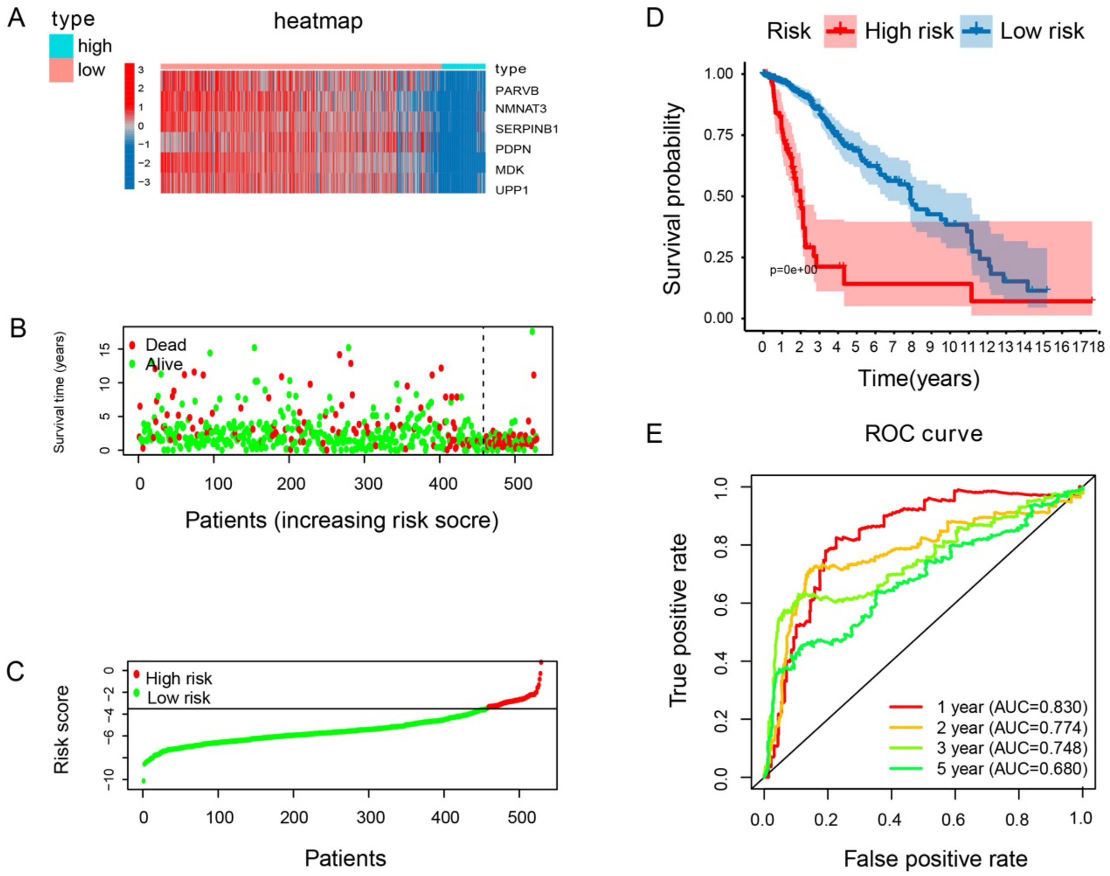

Figure S2 | Validation of DMDGs prognostic model in TCGA LGG set (n=458);

(A) The expression pattern of DMDGs prognostic signature in low and high-risk groups;

(B-C) Survival status and risk score analysis of DMDGs prognostic signature;

(D) Survival analysis of DMDGs prognostic signature;

(E) The ROC curve analysis within 1-, 2-, 3- and 5-year.

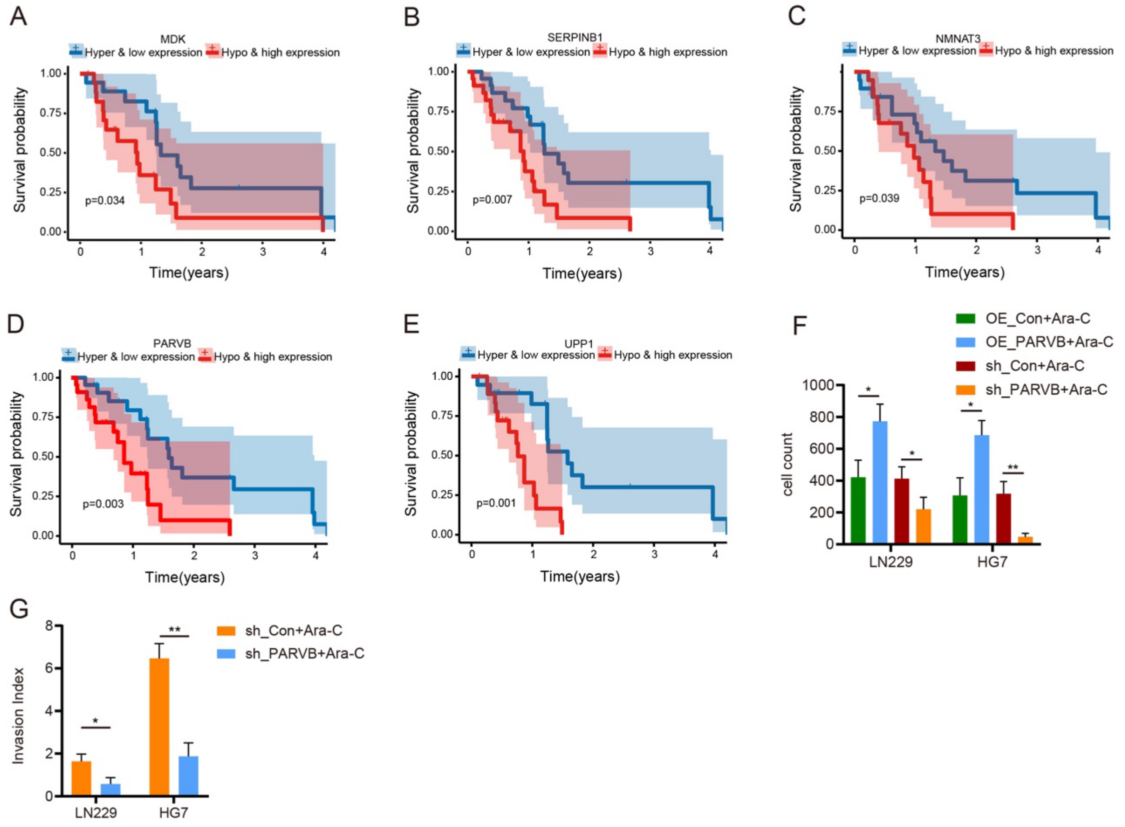

Figure S3| Joint survival analysis and Assessment of migration and invasion independent of proliferation.

(A-E) The hypermethylation low expression genes (MDK, SERPINB1, NMNAT3, PARVB and UPP1) have a significantly better OS than the hypomethylation high expression gene.

(F-G) Ara-C (cytarabine, 3μg/mL) was used to inhibit cell proliferation, and similar results of migration and invasion was observed in LN229 and HG7 cells. The assays were determined from three independent experiments, quantification data are expressed as average ± SD. Significant results are presented as *p < 0.05 and **p < 0.01.

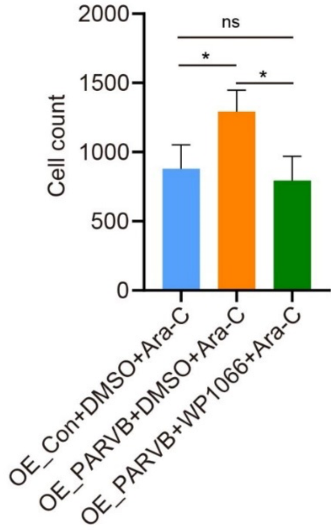

Figure S4 | Assessment of migration and invasion independent of proliferation.

Ara-C (cytarabine, 3μg/mL) was used to inhibit cell proliferation, and similar results of migration was observed in LN229 and HG7 cells. The assays were determined from three independent experiments, quantification data are expressed as average ± SD. Significant results are presented as ns (non-significant), $^*p < 0.05$.
